# Efficacy of non-pharmacological interventions on sleep quality in patients with cancer-related insomnia: a network meta-analysis

**DOI:** 10.3389/fneur.2024.1421469

**Published:** 2024-09-20

**Authors:** Yu Luo, Hua He, Caihong Cao, Ruoxin Xu, Xiaohua Tian, Gufen Jiang

**Affiliations:** ^1^The Second Affiliated Hospital of Hunan University of Traditional Chinese Medicine, Changsha, Hunan, China; ^2^School of Nursing, Hunan University of Traditional Chinese Medicine, Changsha, Hunan, China

**Keywords:** cancer-related insomnia, non-pharmacological intervention, sleep quality, network meta-analysis, cancer

## Abstract

**Objective:**

Despite the widespread application of non-pharmacological therapies in treating cancer-related insomnia, a comprehensive assessment of these methods is lacking. This study aims to compare the efficacy of 11 non-pharmacological interventions for cancer-related insomnia, providing a theoretical basis for clinicians in choosing treatment methods.

**Methods:**

We searched five databases, including the Cochrane Central Register of Controlled Trials, PubMed, Embase, Wiley Library, and Web of Science, for relevant randomized controlled trials. Included studies involved patients diagnosed with cancer-related insomnia, employed non-pharmacological treatments, and reported outcomes using the PSQI and ISI. Bayesian statistical methods were used for the network meta-analysis, and statistical processing was performed using Review Manager 5.4 and Stata 14.0 software. The results were thoroughly analyzed and evaluated, and publication bias was assessed using funnel plot tests.

**Results:**

Our study included 41 randomized controlled trials, comprising 11 different non-pharmacological interventions (3,541 participants), the network analysis identifying Electroacupuncture as the most effective, with a SUCRA value of 92.2% in ISI, this was followed by Professionally administered Cognitive behavioral therapy for insomnia(PCBT-I) and Mindfulness-based cognitive therapy(MBCT), with SUCRA values of 78.4 and 64.1%, respectively. Traditional Cognitive behavioral therapy for insomnia(CBT-I) and VCBT-I showed lower efficacy with SUCRA values of 55.9 and 55.2%, respectively. Exercise interventions and control groups had the lowest efficacy, with SUCRA values of 24.0 and 16.1%. Using PSQI as the outcome measure, Massage therapy ranked highest in improving sleep quality with a SUCRA value of 92.2%, followed by Professionally administered Cognitive behavioral therapy for insomnia (PCBT-I) and Electroacupuncture. League tables indicated significant improvements in sleep outcomes for Electroacupuncture and MT compared to control groups, with Electroacupuncture (EA) showing an MD of −7.80 (95% CI: −14.45, −1.15) and MT an MD of −4.23 (CI: −8.00, −0.46).

**Conclusion:**

Considering both outcome indicators, Electroacupuncture was significantly effective in alleviating the severity of insomnia, while MT was most effective in improving sleep quality. Therefore, in the non-pharmacological interventions for cancer-related insomnia, Electroacupuncture and MT May be particularly effective choices. Future research should further explore the specific mechanisms of action of these interventions and their efficacy in different patient groups.

## 1 Introduction

Cancer, as a prevalent and lethal pathology, has elicited extensive attention due to its escalated incidence and its role as a primary causative factor of mortality in the sphere of global health (1). Cancer-related insomnia is a sleep disorder caused by cancer itself, mood disorders, cancer treatment, or a combination of these factors in patients with malignant tumors. Cancer-related insomnia is defined as difficulty falling asleep, staying asleep, and/or waking up early in the fifth edition of the Diagnostic and Statistical Manual of Mental Disorders. Over 3 months, resulting in severe impairment of daily living. Amongst the myriad challenges inherent in oncological therapy and management, the issue of cancer-associated insomnia is notably acute, this form of insomnia is characterized not merely by a deficiency in sleep duration, but more critically, by a substantial degradation in sleep quality, thereby constituting a severe menace to the physiological and psychological well-being of affected individuals (2). Cancer patients May struggle to fall asleep due to pain, side effects of medications, emotional issues such as anxiety and depression, and physiological changes during treatment (3–5). Surveys indicate that the rate of insomnia in cancer patients is around 60% in global, significantly higher than in the general population, with 20% of these patients experiencing recurrent episodes of insomnia (3–5). Past studies have suggested that this high incidence is attributed to the psychological impact following a cancer diagnosis and the adverse effects of chemotherapy and various treatments, this not only greatly reduces the quality of life for patients during cancer treatment but also exposes them to a high risk of psychological comorbidities such as anxiety and depression (6–8).

At present, the management of cancer-related insomnia primarily encompasses pharmacological and non-pharmacological treatments. In the realm of pharmacotherapy, sedatives and benzodiazepine receptor agonists are commonly employed. However, their usage is often accompanied by numerous side effects, such as daytime somnolence, cognitive and motor functional impairments, dizziness, residual daytime sedation, fatigue, daytime sleepiness, and headache (9–11). Consequently, the exploration of more effective modalities for treating cancer-related insomnia has become an urgent issue. In recent years, some non-pharmacological therapies have been widely supported in the world, with the advantages of few side effects and significant efficacy (12–15). Among these, acupuncture has been widely utilized for insomnia management. Numerous studies have discovered that acupuncture can effectively modulate neurotransmitters such as melatonin, norepinephrine, endorphins, and gamma-aminobutyric acid (GABA), this regulation of neurotransmitters contributes to the improvement of both sleep quality and efficiency (16–19). Additionally, electroacupuncture, which supplements traditional acupuncture with electrical stimulation, has shown even more pronounced effects. Cognitive Behavioral Therapy for Insomnia (CBT-I) and Mindfulness-Based Stress Reduction (MBSR) are also commonly employed non-pharmacological treatments. They have been proven to improve sleep quality in cancer patients. A meta-analysis by Johnson JA et al. (20) highlighted that CBT-I significantly improves sleep efficiency, reduces sleep latency, and alleviates the severity of insomnia. The Study of Fiorentino L (21) suggests that patients can acquire skills to cope with insomnia during CBT-I treatment, preventing or mitigating future insomnia episodes. The formats of CBT-I treatment have diversified, ranging from fully therapist-dependent, partially therapist-dependent, to entirely self-administered. However, a large-scale randomized controlled study by Savard et al. indicated that professional CBT-I (PCBT-I) was most effective in alleviating insomnia, underscoring the importance of face-to-face professional guidance (22). Therefore, healthcare professionals can augment self-administered treatment with flexible professional guidance according to the patient’s specific circumstances to enhance therapeutic outcomes.

Mindfulness-Based Cognitive Therapy (MBCT) represents an integrative therapeutic approach that amalgamates mindfulness meditation techniques, cognitive behavioral therapy, and stress management strategies. This therapy initially utilizes mindfulness meditation techniques to activate neural structures involved in attention regulation and autonomic nervous system control, thereby enhancing relaxation responses, which are beneficial for sleep quality improvement. The synergy of these techniques with cognitive behavioral therapy considerably benefits insomnia improvement, as evidenced in Zhao’s study demonstrating significant effects of MBCT in breast cancer survivors (23). Massage therapy (MT), a widely acclaimed non-pharmacological intervention, derives therapeutic effects through the simple act of touch (24), it improves sleep quality primarily by accelerating blood circulation, aiding digestive system functions, stimulating the lymphatic system, impacting the nervous system, relieving stress, and reducing heart rate and blood pressure. Moreover, massage can induce the release of endorphins, alleviating pain and facilitating a state of relaxation, thereby aiding in sleep quality enhancement (25). The advantages of MT lie in its simplicity of application and the minimal active participation required from patients, resulting in high patient acceptability. Mindfulness-Based Stress Reduction (MBSR) aids patients in recognizing and accepting their current state while developing positive emotional responses and effective stress management strategies to cope with ongoing challenges and events, this approach not only aids in improving sleep symptoms but also positively impacts overall life quality (26).

As non-pharmacological treatments for cancer-related insomnia become increasingly prevalent, comprehensive evaluation and comparison of these methods are crucially important. Currently, there is a scarcity of studies systematically integrating and assessing the efficacy of these disparate treatments. Therefore, our study employs network meta-analysis, allowing direct and indirect comparisons among various non-pharmacological treatments, thereby providing a more comprehensive and systematic assessment of their effects. By comparing 11 different non-pharmacological interventions, we aim to reveal which treatments are most effective for cancer-related insomnia, thus offering a robust scientific foundation for clinicians in selecting non-pharmacological treatments. The application of network meta-analysis not only strengthens the integration of existing evidence but also enhances the flexibility and comprehensiveness of the analysis. Network meta-analysis can handle data from different studies, even when there are variations in design and execution. Furthermore, this method provides probabilistic assessments of treatment effects, enabling more accurate judgments of the relative efficacy of various non-pharmacological interventions. Therefore, through the network meta-analysis in this study, we hope to provide a clear and scientific guide for non-pharmacological treatments of cancer-related insomnia, ultimately improving the sleep quality and overall therapeutic outcomes for cancer patients.

## 2 Methods

### 2.1 Search strategy and study selection

The research team systematically searched five databases: Cochrane Central Register of Controlled Trials, PubMed, Embase, Wiley, and Web of Science. The search covered all entries from the inception of these databases to October 2023, with no language restrictions. The search strategy primarily combined subject headings with free-text terms, using Boolean operators for linkage. Key search terms included “cancer,” “tumor,” “cancer-related insomnia,” “sleep pattern disorders,” “sleep disturbances,” “non-pharmacological intervention,” “cognitive behavioral therapy,” “mindfulness-based stress reduction,” “acupuncture,” “randomized controlled trials,” and other keywords relevant to the topic.

This study was conducted and reported in strict adherence to the Preferred Reporting Items for Systematic Reviews and Meta-Analyses for Network Meta-Analysis (PRISMA-NMA) guidelines. All analyses were based on previously published literature, thus obviating the need for ethical approval or patient consent. The meta-analysis has been registered with PROSPERO, registration number CRD42023465232^1^. Two researchers independently screened the literature according to predefined inclusion and exclusion criteria. Initially, bibliographic records retrieved from searches were imported into the reference manager EndNote 20. After systematic duplication checks, redundant publications were removed, then the titles and abstracts of the retrieved articles were thoroughly read to filter out those not meeting the criteria. Finally, full texts were obtained and reviewed for determination. Following the independent screening by the two researchers, the selected studies were cross-checked. If a consensus could not be reached, a third party with extensive experience intervened to adjudicate the disagreement.

### 2.2 Inclusion and exclusion criteria and data extraction

Two researchers initially identified relevant literature using the designed search strategy, the selection of studies for inclusion and the extraction of data were rigorously conducted in accordance with the PICOS (Population, Intervention, Comparison, Outcomes, and Study design) criteria, ultimately including randomized controlled trials that met these standards.

### 2.3 Population

① Patients aged ≥18 years old; ② Participants in this study were patients diagnosed with cancer-related insomniar; ③ Patiens insomnia developed after the diagnosis of cancer. Insomnia occurred at least 3 nights/week and lasted for at least 3 months, meeting the diagnostic and Statistical Manual of Mental Disorders (5th edition) diagnostic criteria for transient insomnia; ④ Insomnia severity index score was more than 8 points; ⑤ Pittsburgh Sleep Quality Index (PSQI) score > 6.

### 2.4 Interventions

The non-pharmacological interventions for cancer-related insomnia included: CBT-I, MBSR, Brief BBTI, Electroacupuncture, Acupuncture, ICBT-I, PCBT-I, MBCT, Exercise, HIIT, and Massage Therapy.

#### 2.4.1 Comparisons

Different non-pharmacological intervention groups were compared either with control groups or against each other. Control groups primarily consisted of standard care or no intervention.

#### 2.4.2 Outcomes

The primary outcome measure in this study was sleep quality. Considering the variety of available tools for assessing sleep quality, however, the commonly used tools for assessing sleep quality are the Insomnia Severity Index and the Pittsburgh Sleep Quality Index. Therefore, trials using the Insomnia Severity Index (ISI) and the Pittsburgh Sleep Quality Index (PSQI) as outcome measures were included in this study.

#### 2.4.3 Study design

All studies included in this network meta-analysis were randomized controlled trials, published in peer-reviewed journals or online, with no restrictions on the publication date. Studies with missing content, those for which full texts could not be obtained, or those still in the research phase were excluded.

The developed search strings were used to search multiple databases, and the retrieved literature entries were imported into EndNote for deduplication. Following the predetermined inclusion and exclusion criteria, two researchers independently screened the titles and abstracts of the preliminary search results. Full-text readings were conducted when titles and abstracts were insufficient for decision-making. In the process of literature retrieval and screening, studies with disputes were reviewed by a third, experienced individual after the two researchers provided their explanations.

### 2.5 Data extraction and quality assessment

The general information extracted from the included randomized controlled trials (RCTs) encompassed the author(s) of the study, year of publication, country, and patient characteristics (age, gender, type of surgery), as well as the interventions employed in both the trial and control groups. The primary outcomes of the included trials were compared with respective control groups to evaluate the efficacy of different non-pharmacological interventions in treating cancer-related insomnia. All included studies used either the ISI or the PSQI as assessment tools. Therefore, we extracted the ISI scores or PSQI values from the last observation point to assess the improvement in cancer-related insomnia.

Two researchers independently conducted a quality assessment of each included RCT using the Cochrane Risk of Bias 1 (ROB1) tool. The ROB1 covers seven domains: random sequence generation, allocation concealment, blinding of participants and personnel, blinding of outcome assessment, incomplete outcome data, selective reporting, and other biases. The risk of bias in these seven domains is classified as “high risk,” “unclear risk,” or “low risk.” Each researcher independently assessed the risk of bias for each domain, and the assessments were then entered into Review Manager 5.4 software for ROB1 evaluation. In instances where there was disagreement between the two researchers, a third researcher intervened to make a judgment.

### 2.6 Statistical analysis

In this study, we conducted a Network Meta-Analysis (NMA) using STATA 14 to synthesize and compare the relative effects of three or more treatment methods. This approach allowed us to integrate both direct and indirect evidence, offering a comprehensive assessment of treatment effectiveness. This approach was used to comprehensively compare the efficacy of 11 non-pharmacological interventions for cancer-related insomnia. To ensure accuracy and reliability in our analysis, we rigorously assessed the transitivity assumption, which examines the similarity of clinical and methodological characteristics, including patient populations and experimental designs, across studies. Additionally, we employed the node-splitting method to test for consistency between direct and indirect evidence within the network, with a *p < 0.05* indicating significant inconsistency. The quality assessment of RCT literature was performed using RevMan 5.4. The statistical effect size was represented by the Mean Difference (MD), accompanied by a 95% Confidence Interval (CI). The Surface Under the Cumulative Ranking Curve (SUCRA) was used to evaluate and rank the efficacy of each treatment comprehensively, with higher SUCRA values indicating better treatment outcomes. To assess the robustness of the NMA results, we recalculated the ranking probabilities by excluding studies with a medium to high risk of bias and conducting sensitivity analyses. If the analysis results showed no significant statistical differences, we considered the NMA results to be valid.

## 3 Results

### 3.1 Characteristics and quality of included studies

**Figure 1:**
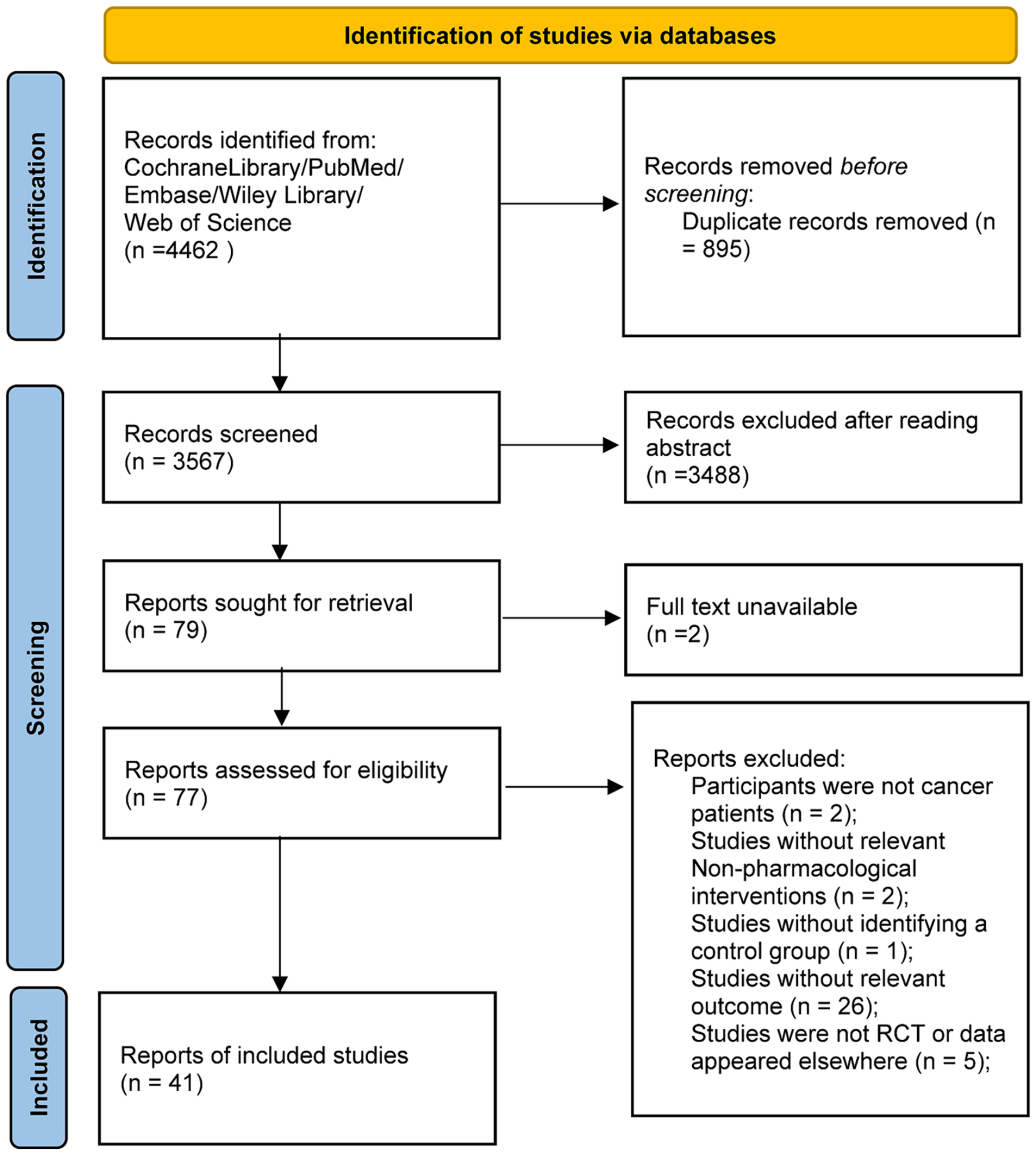
Literature screening flow chart.

A total of 4,462 articles were identified through the search, with 895 duplicates removed, after an initial review of titles and abstracts, 3,488 articles were excluded for not meeting the inclusion criteria. An additional 38 articles were excluded, consequently, 41 randomized controlled trials were ultimately included in the study, as shown in Figure 1.

The included 41 studies encompassed 3,541 participants, with 1924 assigned to non-pharmacological intervention groups and 1,617 to control groups. The included studies covered the period from 2008 to 2022 and were from multiple regions. Nineteen studies were from the USA, 9 studies from China, 6 studies from Canada, 2 studies from Denmark, 1 study from Germany, 1 study from Sweden, 1 study from India, 1 study from Belgium, and 1 study from Iran. Breast cancer was the most common cancer type, involving 23 studies, while others included lung, ovarian, and prostate cancers, among others, with 15 studies not specifying the cancer type. Details are presented in Table 1. In terms of quality assessment, 40 studies reported the method of random sequence generation, and 31 described allocation concealment methods. The quality risk assessment revealed high bias risk in blinding in 8 studies, in missing data in 1 study, and in selective reporting in 1 study. The other studies were of relatively high quality, as shown in Supplementary Figures S1, S2.

**Table 1:** General characteristics of the included literature.

Author	Year	Country	Sample Size		Age(Mean ± SD)		Gender		Surgery history		Intervention		Cancer type	Outcomes
			C	E	C	E	C	E	C	E	C	E		
Amidi (50)	2022	Denmark	54	77	53.5 ± 8.9	54.0 ± 7.8	54	77	/	/	Control	CBT-I	Breast cancer	PSQI
Applebaum (51)	2020	America	6	7	/	/	/	/	/	/	Acupuncture	CBT-I	Cancer	ISI
Arem (52)	2019	America	11	14	52.8 ± 7.1	52.9 ± 9.8	11	14	/	/	Mind–Body	CBT-I	Breast cancer	ISI/PSQI
Bao (53)	2021	America	24/24	27	57.3/62.7	60.3	21/19	20	/	/	Control/Sham acupuncture	Real acupuncture	Cancer	ISI
Casault (54)	2015	Canada	18	20	57.0 ± 9.4	56.9 ± 10.8	16/18	19/20	/	/	Routine care	CBT-I	Cancer	ISI
Chen (55)	2013	China	47	49	44.7 ± 9.7	45.3 ± 6.3	47	49	46	49	Control	Qigong	Breast cancer	PSQI
Cheung (56)	2022	China	15	15	58.93 ± 11.11	61.8 ± 9.92	12	12	/	/	Control	Acupressure	Cancer	PSQI
Dean (57)	2020	America	14	16	65.86 ± 8.08	65.63 ± 7.26	9/14	10/16	/	/	Control	CBT-I	Lung cancer	ISI/PSQI
Dirksen (58)	2008	America	41	40	59.2 ± 10.7	57.2 ± 9.9	41	40	10/41	8/40	Routine care	CBT-I	Breast cancer	ISI
Feng (59)	2011	China	40	40	63.60 ± 4.26	63.80 ± 5.47	13/40	14/40	/	/	Control	Acupuncture	Cancer	PSQI
Fiorentino (60)	2010	America	10	11	/	/	/	/	/	/	Routine care	CBT-I	Breast cancer	ISI/PSQI
Garland (61)	2014	America	40	32	58.73 ± 10.46	60.33 ± 12.21	32/40	20/32	35/40	24/32	CBT-I	MBSR	Cancer	ISI/PSQI
Garland (62)	2016	Canada	65	65	/	/	/	/	/	/	CBT-I	Acupuncture	Cancer	ISI
Garland (13)	2019	America	80	80	60.7 ± 12.0	62.3 ± 11.4	48/80	43/80	52/80	63/80	CBT-I	Acupuncture	Cancer	ISI/PSQI
Höxtermann (63)	2021	Germany	26	26	54.8 ± 8.3	56.58 ± 7.9	26	26	/	/	Control	Acupuncture	Breast cancer	PSQI
Huang (64)	2019	China	78	81	48.32 ± 7.90	48.32 ± 7.90	78	81	78	81	Control	Exercise	Breast cancer	PSQI
Irwin (65)	2017	America	45	45	60.0 ± 9.3	59.6 ± 7.9	45	45	4/45	6/45	CBT-I	Tai Chi	Breast cancer	PSQI
Kashani (24)	2014	Iran	30	30	/	/	30	30	/	/	Control	Massage therapy	Breast cancer	PSQI
Lee (3)	2022	America	8/6	8	61.38/62.33	57.63	5/8and1/6	6/8	/	/	Control/sham electroacupuncture	Electroacupuncture	Cancer	ISI/PSQI
Lengacher (66)	2015	America	41	38	58.0 ± 10.2	56.1 ± 9.1	41	38	41	38	Control	MBSR	Breast cancer	PSQI
Liu (67)	2022	China	34	38/36/36	51.41 ± 10.10	48.58 ± 8.48/53.06 ± 8.29/52.67 ± 8.19	32/34	38/36/36	33/34	37/30/34	Control	MBSR/Acupressure/MBSR combined with acupressure	Breast cancer	PSQI
Mao (68)	2014	America	23/22	22	60.6 ± 8.2/60.9 ± 6.5	57.5 ± 10.1	/	/	/	/	Control/sham acupuncture	Real acupuncture	Breast cancer	PSQI
Matthews (69)	2014	America	32	28	52.85 ± 7.75	52.17 ± 6.86	32	28	/	/	Control	CBT-I	Breast cancer	ISI
Mercier (70)	2018	Canada	20	21	56.6 ± 10.2	57.6 ± 9.3	16/20	16/21	16/20	16/21	Exercise	CBT-I	Cancer	ISI
Mustian (71)	2013	America	37	34	53.8 ± 11.17	50.9 ± 7.91	37	34	/	/	Control	BBT-CI	Breast cancer	PSQI
Oswald (72)	2022	America	15	15	59.98 ± 9.58	56.90 ± 8.91	15	15	/	/	Control	CBT-I	Breast cancer	ISI
Palesh (73)	2018	America	37	34	53.8 ± 11.17	50.9 ± 7.91	37	34	/	/	Control	BBT-CI	Breast cancer	ISI
Piraux (74)	2021	Belgium	26	27/25	71.9 ± 8.1	67.4 ± 8.9/67.9 ± 7.1	/	/	/	/	Control	HIIT/RES	Prostate cancer	ISI/PSQI
Rao (75)	2017	India	46	45	50.2 ± 9.2	48.9 + 9.1	46	45	10/46	3/45	Control	Yoga	Breast cancer	ISI
Ritterband (76)	2012	America	14	14	59.6 ± 12.3	53.7 ± 10.8	10/14	14	/	/	Control	CBT-I	Cancer	ISI
Savard (22)	2014	Canada	81	81/80	55.4 ± 8.8	52.6 ± 8.9/55.3 ± 8.7	81	81/80	/	/	Control	PCBT-I/CBT-I	Breast cancer	ISI
Savard (77)	2016	Canada	80	81/81	55.4 ± 8.8/52.6 ± 8.9	55.3 ± 8.7	/	/	/	/	Control/PCBT-I	VCBT-I	Breast cancer	ISI
Savard (78)	2021	Canada	59	118	54.8 ± 11.1	55.5 ± 10.0	48/59	103/118	49/59	106/118	StanCBT-I	CBT-I (StepCBT-I)	Cancer	ISI
Sveen (79)	2021	Sweden	11	10	45.6 ± 5.5	49.9 ± 5.8	7/11	7/10	/	/	Control	CBT-I	Cancer	ISI
Yang (15)	2021	America	35	35	62.4 ± 10.5	60.8 ± 11.2	25/35	18/35	26/35	27/35	CBT-I	Acupuncture	Cancer	ISI
Zachariae (80)	2018	Denmark	122	133	50.1 ± 8.9	50.3 ± 8.8	122	133	33.9%	31.60%	Control	CBT-I	Breast cancer	ISI/PSQI
Zhang (81)	2023	China	69	69	52.7 ± 8.3	51.7 ± 9.6	69	69	/	/	Sham acupuncture	Active acupuncture	Breast cancer	PSQI
Zhang (82)	2021	China	15	15	52.7 ± 6.3	52.5 ± 8.9	15	15	/	/	Control	Acupuncture	Breast cancer	ISI/PSQI
Zhang (83)	2018	China	36	36	/	/	36	36	/	/	Control	CBT-I	Ovarian cancer	ISI/PSQI
Zhao (23)	2020	China	68	68	53.29 ± 6.50	52.79 ± 6.54	/	/	68	68	Control	MBCT-I	Breast cancer	ISI
Zhi (84)	2021	America	20	21	62.3	60	/	/	/	/	Control	Yoga	Cancer	ISI

### 3.2 NMA results

In this study, funnel plots were employed to explore potential publication bias. The horizontal axis of the funnel plot represented the Mean Difference (MD), while the vertical axis reflected the Standard Error (SE) of each study. Theoretically, studies with larger sample sizes and higher precision should cluster at the top of the funnel plot, while those with smaller sample sizes and lower precision scatter at the bottom. The adjusted funnel plots exhibited relative symmetry on both sides, although some study points deviated from the main distribution, indicating possible publication bias (Supplementary Figures S3, S4).

**Figure 2:**
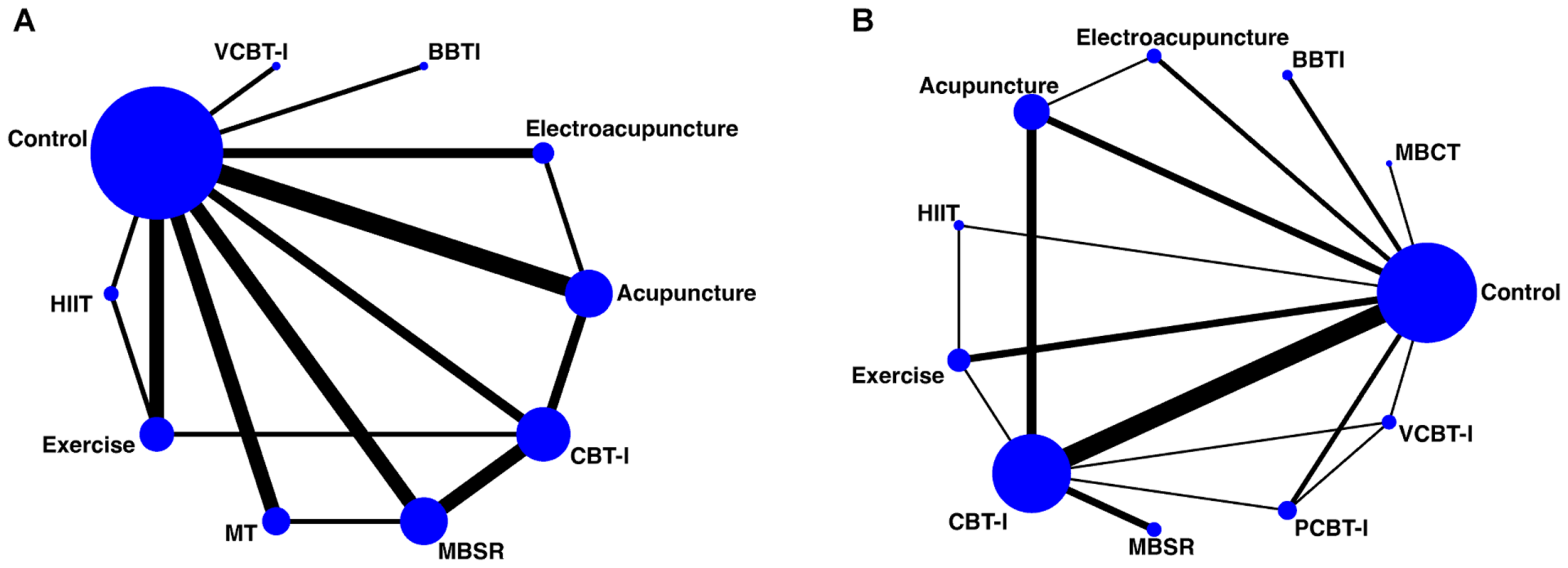
Network diagram illustrating the comparative relationships among non-pharmacological interventions [**(A)** ISI and **(B)** PSQI].

The network diagrams primarily displayed the main evidence for the included non-pharmacological interventions. Each node represented a different non-pharmacological intervention, with the size depending on the number of participants involved in that intervention. These nodes related to lines of varying thickness, indicating the presence and quantity of direct evidence comparisons between different non-pharmacological interventions. The diagrams, comprising multiple closed loops, revealed multiple direct evidence comparisons among the non-pharmacological therapies (Figure 2).

The results of the network meta-analysis, using the ISI as the evaluation standard, are presented in the SUCRA plots, ranking the 11 non-pharmacological therapies. Electroacupuncture showed the best performance with a SUCRA value of 92.2%, indicating its significant advantage in alleviating insomnia. This was followed by PCBT-I and MBCT with SUCRA values of 78.4 and 64.1%, respectively, suggesting their efficacy in treating insomnia. Traditional CBT-I and VCBT-I had SUCRA values of 55.9 and 55.2%, respectively, which, although high, were slightly lower compared to the methods. Conversely, Exercise interventions and the Control group showed relatively lower efficacy, with SUCRA values of 24.0 and 16.1%, respectively. Similarly, when using the PSQI as the outcome measure for SUCRA analysis, MT ranked highest with a SUCRA value of 92.2%, demonstrating its superior efficacy in improving sleep quality. PCBT-I and Electroacupuncture followed closely with SUCRA values of 78.4 and 64.1%, respectively. MBSR and CBT-I also showed efficacy in enhancing sleep quality with SUCRA values of 55.9 and 55.2%, respectively. VCBT-I and the Control group had more limited effects, with SUCRA values of 24.0 and 16.1% (Tables 2, 3 and Supplementary Figures S5, S6).

**Table 2:** Comparative efficacy ranking of interventions for sleep quality improvement based on ISI scores.

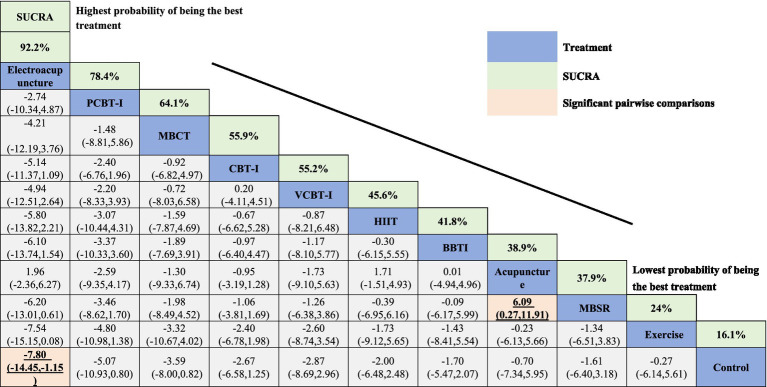

**Table 3:** Comparative efficacy ranking of interventions for sleep quality improvement based on PSQI scores.

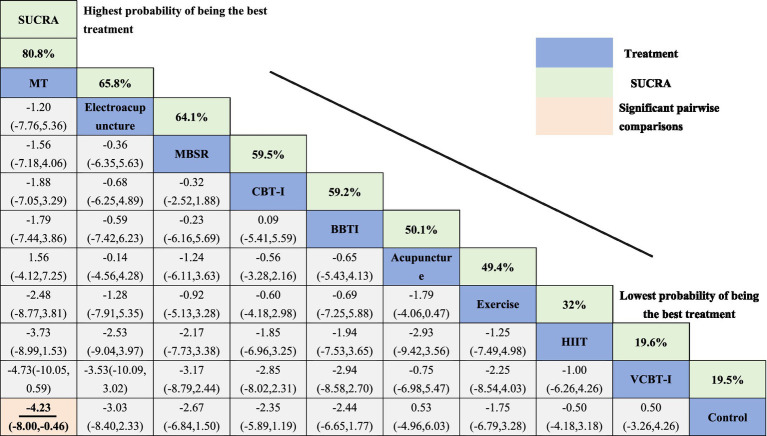

Direct and indirect analyses of the interventions, based on the league tables with ISI as the outcome measure, indicated statistically significant differences in comparisons between Electroacupuncture and Control, as well as between Acupuncture and MBSR. The potential impact indicators were represented with a 95% CI, with Electroacupuncture showing (MD = –7.80, CI: −14.45, −1.15), as seen in Table 2. In the league tables with PSQI as the outcome measure, the comparison between MT and Control was statistically significant, with MT (MD = -4.23, CI: −8.00, −0.46), as seen in Table 3.

## 4 Discussion

At present, NMAs focusing on non-pharmacological treatments for cancer-related insomnia are relatively scarce, and existing studies do not comprehensively cover all data sources. Hence, to assess the relative efficacy of various non-pharmacological therapies more accurately, a broader integration of evidence is necessary. This study, by including a diverse range of non-pharmacological treatments and conducting comprehensive direct or indirect comparisons of their efficacy, provides a solid theoretical foundation for clinicians in selecting non-pharmacological intervention strategies for cancer-related insomnia.

While the overall survival rates of cancer survivors have improved, many continue to face a variety of life-quality-impacting complications post-treatment (27). Among these complications, insomnia stands out prominently and is considered one of the most common post-cancer treatment complications. It is estimated that untreated chronic insomnia not only exacerbates fatigue, depressive symptoms, and pain in patients but May also increase the risk of cancer recurrence and even lead to cognitive decline (28, 29). Although traditional pharmacological treatments are effective, their wide range of adverse reactions limits their broad application. In contrast, an increasing body of research demonstrates that non-pharmacological interventions, such as CBT, MBSR, and acupuncture, can effectively alleviate symptoms of cancer-related insomnia and significantly improve sleep quality (30–32). Therefore, non-pharmacological therapies, as important alternative or complementary treatment modalities, should receive more attention and research in the sleep management of cancer survivors.

This network meta-analysis aimed to assess the efficacy of non-pharmacological interventions for insomnia in cancer patients. Comprehensive analysis of the current randomized controlled trials revealed that among the various non-pharmacological interventions considered, only Electroacupuncture and MT demonstrated statistically significant improvements in insomnia symptoms. Electroacupuncture showed superior performance, consistently effective in both reducing the severity of insomnia and enhancing sleep quality. Acupuncture, a traditional Chinese medical treatment, has been widely applied in improving the quality of life for cancer patients. In Europe, approximately one-third of cancer patients have used acupuncture. Additionally, about 59% of National Cancer Institutes (NCI) have incorporated acupuncture into their cancer treatment management programs (33–35), indicating widespread recognition of its efficacy. Electroacupuncture, an advanced form of acupuncture involving electrical stimulation of acupoints, has been proven to have similar or even superior effects compared to traditional acupuncture (36). Many studies are exploring the mechanisms of electroacupuncture in treating insomnia. On one hand, it is believed to regulate heart rate variability, stabilize blood pressure, and modulate sympathetic nerve activity. Research by Liu Ping and others found that electroacupuncture could alleviate abnormal excitation in the sympathetic adrenal medullary system of insomnia rats, potentially underlying its efficacy in treating cancer-related insomnia (37, 38). On the other hand, electroacupuncture can improve insomnia by inhibiting the activation of the hypothalamic–pituitary–adrenal (HPA) axis, increasing levels of GABA and GABA(A) receptors, and enhancing the production and secretion of melatonin (39–41). Additionally, it can alleviate insomnia by reducing pain caused by surgery and treatment (42, 43). Therefore, electroacupuncture effectively improves sleep quality by alleviating physical (fatigue, pain) and psychological (anxiety, depression) comorbidities associated with cancer-related treatments.

Moreover, CBT-I, an effective adjunctive treatment, has also shown promising results in alleviating cancer-related insomnia. Recognized as the gold standard treatment for insomnia, CBT-I involves sleep hygiene, stimulus control, sleep restriction, cognitive therapy, and relaxation training, with its efficacy widely acknowledged (12). Meta-analyses by Johnson and others have confirmed the efficacy of CBT-I in improving sleep quality in cancer survivors, consistent with our study’s findings (20). CBT-I’s applicability extends beyond clinical settings, with its diverse formats, such as individual, group, face-to-face, and remote (telephone or video), many of which have been proven effective in research. PCBT-I and VCBT-I, derivatives of CBT-I, were compared by Savard for their efficacy in cancer-related insomnia, showing PCBT-I’s superiority over VCBT-I, although the difference was not statistically significant, aligning with our study’s findings (44). However, from a cost-efficacy perspective, VCBT-I is more advantageous than PCBT-I, making it a valuable option for patients with limited resources. With the diversification of CBT-I formats, healthcare professionals can tailor treatments to patients’ circumstances. MBSR, a commonly used complementary therapy, has shown positive effects in treating symptom clusters in cancer patients, including breast and lung cancer (45). Our study also found MBSR effective in alleviating insomnia in cancer patients. Meta-analyses indicate that MBSR can effectively alleviate anxiety and depression in lung cancer patients, reducing psychosomatic symptoms (46). However, there is some debate regarding the timing of MBSR interventions (46). Bisseling (47) suggest that MBSR interventions during breast cancer treatment allow patients to quickly apply learned skills to cope with adverse reactions, but interventions during this period consume considerable time and energy Conversely, some studies argue that implementing MBSR post-treatment might be less effective due to accumulated fear and anxiety (48), therefore, the timing of MBSR interventions is crucial. Our study included a limited number of MBSR studies with varying cancer types and intervention timings, potentially leading to efficacy differences across different outcomes. Future MBSR research should include an assessment of patients’ psychological states, allowing patients more autonomy in practice choices, and further validating the therapy’s efficacy. Exercise has also been proven effective in mitigating adverse reactions during cancer treatment. Mishra (49) indicates that exercise-related interventions are beneficial for improving the quality of life in cancer survivors, yet the specifics of exercise (mode, intensity, frequency, duration, timing) should be tailored according to the type of cancer and treatment status. Our study grouped Yoga, Taichi, and resistance training as exercise, which May have introduced heterogeneity due to the factors mentioned above. Hence, the efficacy of exercise requires further exploration in future research.

Based on the findings of this network meta-analysis, Electroacupuncture stands out as the most effective treatment for cancer-related insomnia. To maximize therapeutic outcomes, it is recommended that professional acupuncturists, while administering Electroacupuncture, should be complemented by healthcare professionals who strengthen health education and sleep management guidance for cancer patients. This integrated approach can further improve patients’ sleep quality. While Electroacupuncture requires professional execution and May have certain limitations in clinical application, it remains a prominent treatment option. On the other hand, CBT-I, as an effective method for treating insomnia, is becoming increasingly flexible and diverse in its application. CBT-I can be implemented in various formats, ranging from fully therapist-dependent to partially therapist-dependent, or even entirely self-administered. This flexibility allows healthcare providers to tailor the treatment approach based on the specific circumstances and needs of the patient. Therefore, when addressing different patients, healthcare professionals should thoroughly consider the characteristics and adaptability of each treatment modality to ensure the provision of the best possible therapeutic plan for each patient.

### 4.1 Strengths and limitations of the study

The most significant strength of this study is the integration of a variety of non-pharmacological interventions. Previous research in this area often focused on a limited range of non-pharmacological interventions, and our study addresses this gap by providing a more comprehensive analysis. Additionally, the lack of network meta-analyses on non-pharmacological interventions for cancer-related insomnia underlines the necessity of this study. However, the study also has its limitations. Firstly, the use of two different scales for outcome assessment May lead to variability in results, owing to differences in their composition, scoring criteria, and sensitivity. Secondly, many of the studies did not mention blinding methods or had a high risk of bias in blinding, which could potentially skew the results and affect the evaluation of efficacy. Thirdly, this review focused solely on cancer patients with sleep problems, meaning the findings regarding the efficacy of interventions in improving the severity of insomnia and sleep quality are only applicable to this population. Lastly, the limited data provided in some papers meant that only a few studies were available for certain interventions, restricting the ability to fully investigate these treatments and draw conclusive results. Moreover, the exclusion of studies that did not contain the specified outcome measures May have omitted relevant research. Future research should incorporate larger sample sizes, multi-center, double-blind RCTs to validate the conclusions and minimize bias to the greatest extent possible.

## 5 Conclusion

This study synthesized various data and concluded that among the non-pharmacological interventions for cancer-related insomnia, Electroacupuncture emerged as the most effective method, followed closely by CBT-I. These findings provide crucial guidance for clinical healthcare professionals in treating patients with cancer-related insomnia. Given its significant efficacy, Electroacupuncture should be more widely implemented in clinical practice to aid cancer patients in improving their sleep quality. Concurrently, CBT-I, as an effective non-pharmacological treatment, should also be considered a vital adjunctive therapy option. Future research should delve further into optimizing the application and efficacy of these interventions, aiming to provide the best personalized treatment plans for cancer patients. However, there are still some shortcomings in this study. Because this study only uses PSQI and ISI as assessment tools for insomnia, and does not use sleep diary to evaluate the continuous changes after treatment, it is necessary to supplement them for evaluation in future research.

## Data Availability

The datasets presented in this study can be found in online repositories. The names of the repository/repositories and accession number(s) can be found in the article/Supplementary material.

## Footnotes

^1^ https://www.crd.york.ac.uk/
