# Supplementary material for: Efficacy of non-pharmacological interventions on sleep quality in patients with cancer-related insomnia: a network meta-analysis
Source: Front Neurol. 2024 Sep 20;15:1421469. doi: 10.3389/fneur.2024.1421469 (PMC11449704; doi:10.3389/fneur.2024.1421469)
Supplement: Supplementary file 1 [file Data_Sheet_1.docx]

**Supplementary material**

**Search strategy**

**1|PubMed (2307 items)**

((Cancer[Title/Abstract] OR tumor[Title/Abstract] OR neoplasms[Title/Abstract]) AND (insomnia[Title/Abstract] OR sleep disorder[Title/Abstract] OR sleep disturbance[Title/Abstract])) AND (intervention[Title/Abstract] OR treatment[Title/Abstract] OR therapy[Title/Abstract] OR cure[Title/Abstract] OR Cognitive behavioral Therapy[Title/Abstract] OR Mindfulness-based stress reduction[Title/Abstract] OR acupuncture[Title/Abstract])

**2|Cochrane Library (1162 items)**

Cancer OR tumor OR neoplasms in Record Title AND insomnia OR sleep disorder OR sleep disturbance in Abstract AND intervention OR treatment OR therapy OR cure OR Cognitive behavioral Therapy OR Mindfulness-based stress reduction OR acupuncture in Abstract - in Trials, Clinical Answers (Word variations have been searched)

**3|Wiley Library (10 items)**

"Cancer OR tumor OR neoplasms" in Abstract and "insomnia OR sleep disorder OR sleep disturbance" in Abstract and "intervention OR treatment OR therapy OR cure OR Cognitive behavioral Therapy OR Mindfulness-based stress reduction OR acupuncture"

**4|Web of Science (416 items)**

((TI=(Cancer OR tumor OR neoplasms)) AND TI=( insomnia OR sleep disorder OR sleep disturbance)) AND TI=(intervention OR treatment OR therapy OR cure OR Cognitive behavioral Therapy OR Mindfulness-based stress reduction OR acupuncture)

**5|Embase (577 items)**

('cancer':ab,ti OR 'tumor':ab,ti OR 'neoplasms':ab,ti) AND (insomnia:ab,ti OR 'sleep disorder':ab,ti OR 'sleep disturbance':ab,ti) AND (intervention:ab,ti OR 'treatment':ab,ti OR 'cure':ab,ti OR 'cognitive behavioral therapy':ab,ti OR 'mindfulness-based stress reduction':ab,ti) AND [randomized controlled trial]/lim


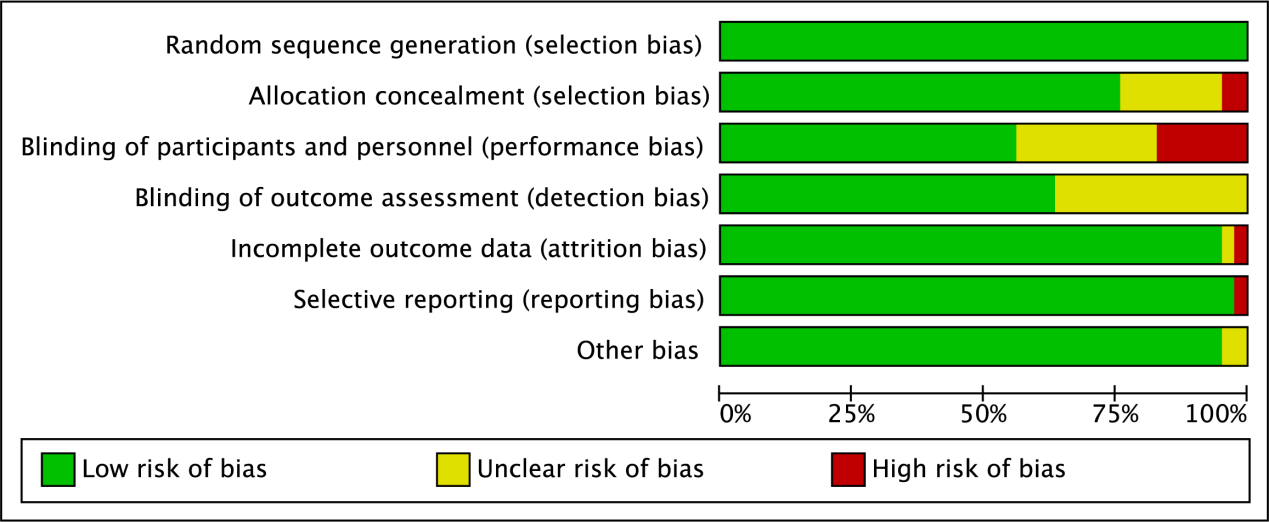


Figure S1 Risk of bias graph


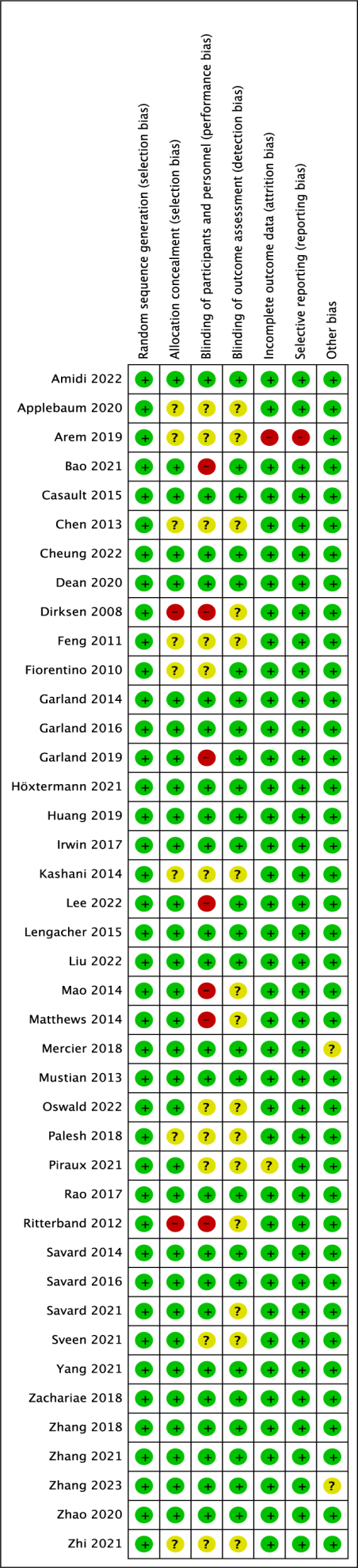


Figure S2 Risk of bias summary


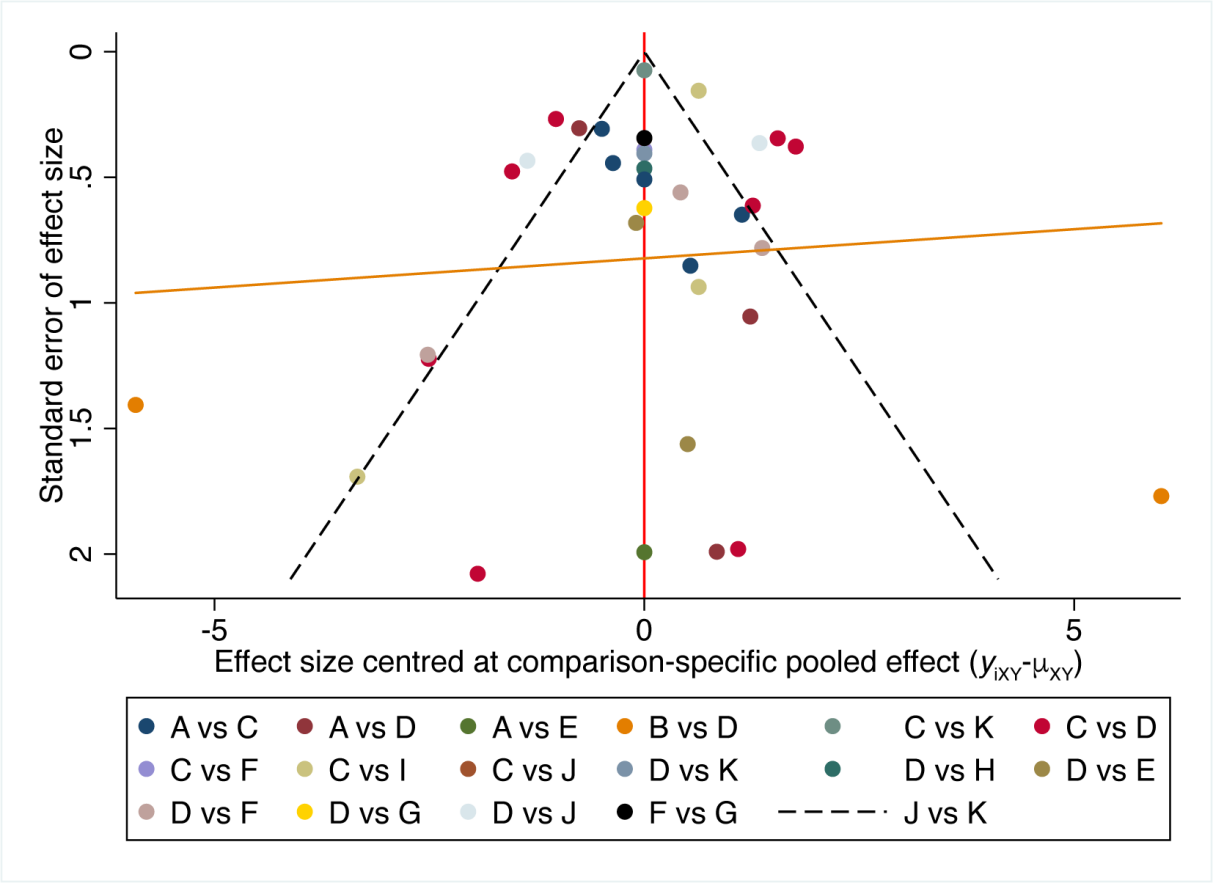


**Figure S3 Funnel Plot for ISI Assessing Publication Bias**


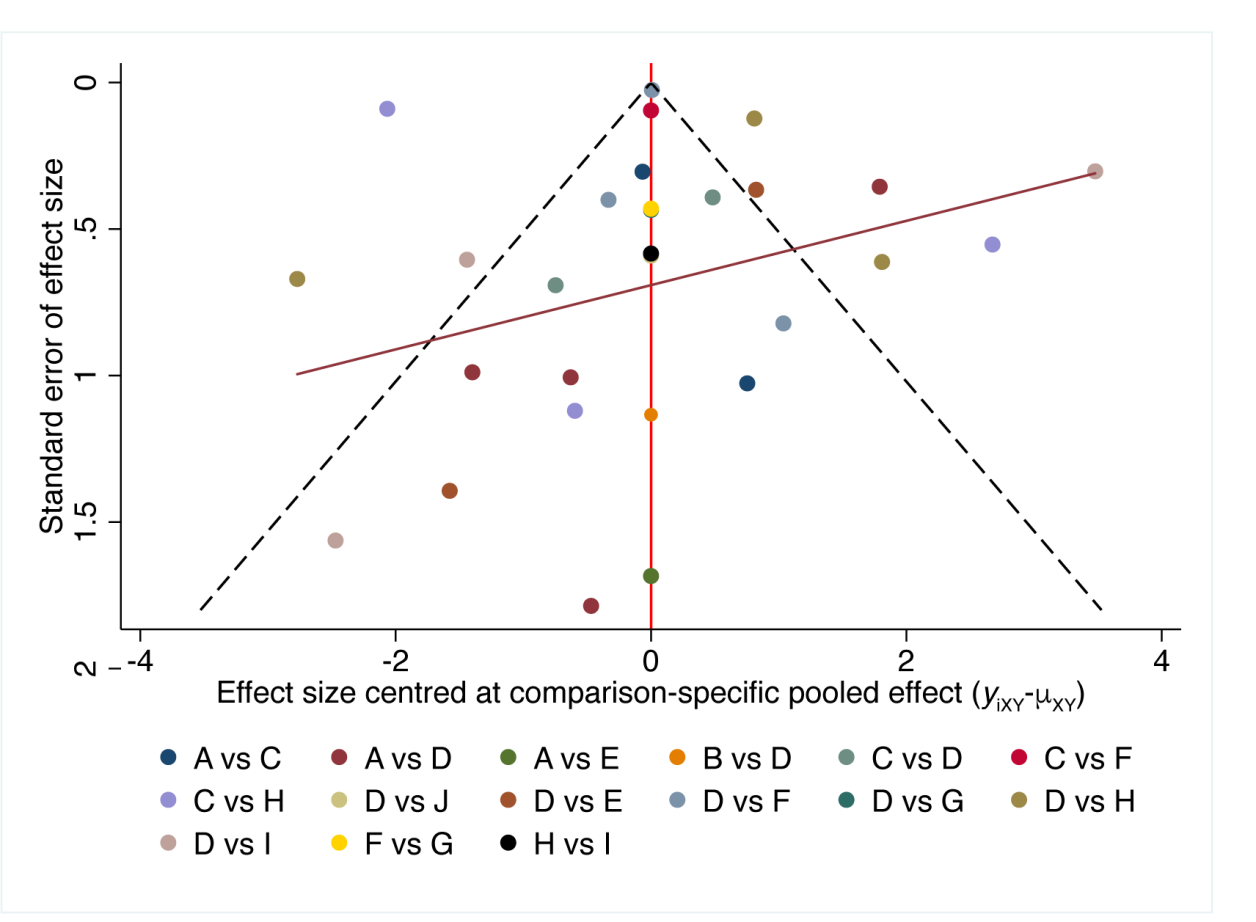


**Figure S4 Funnel Plot for PSQI Assessing Publication Bias**


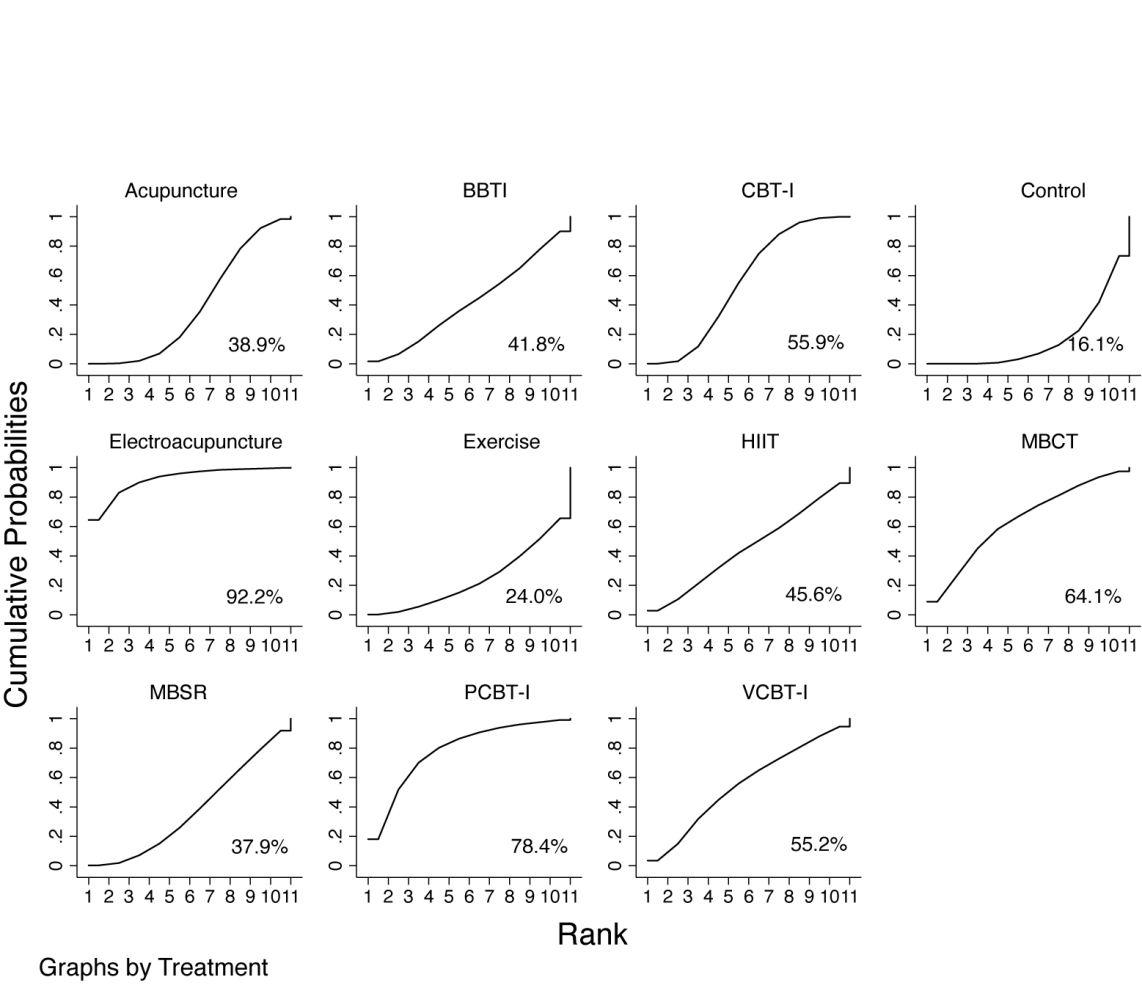


**Figure S5 SUCRA Ranking Plot for ISI**


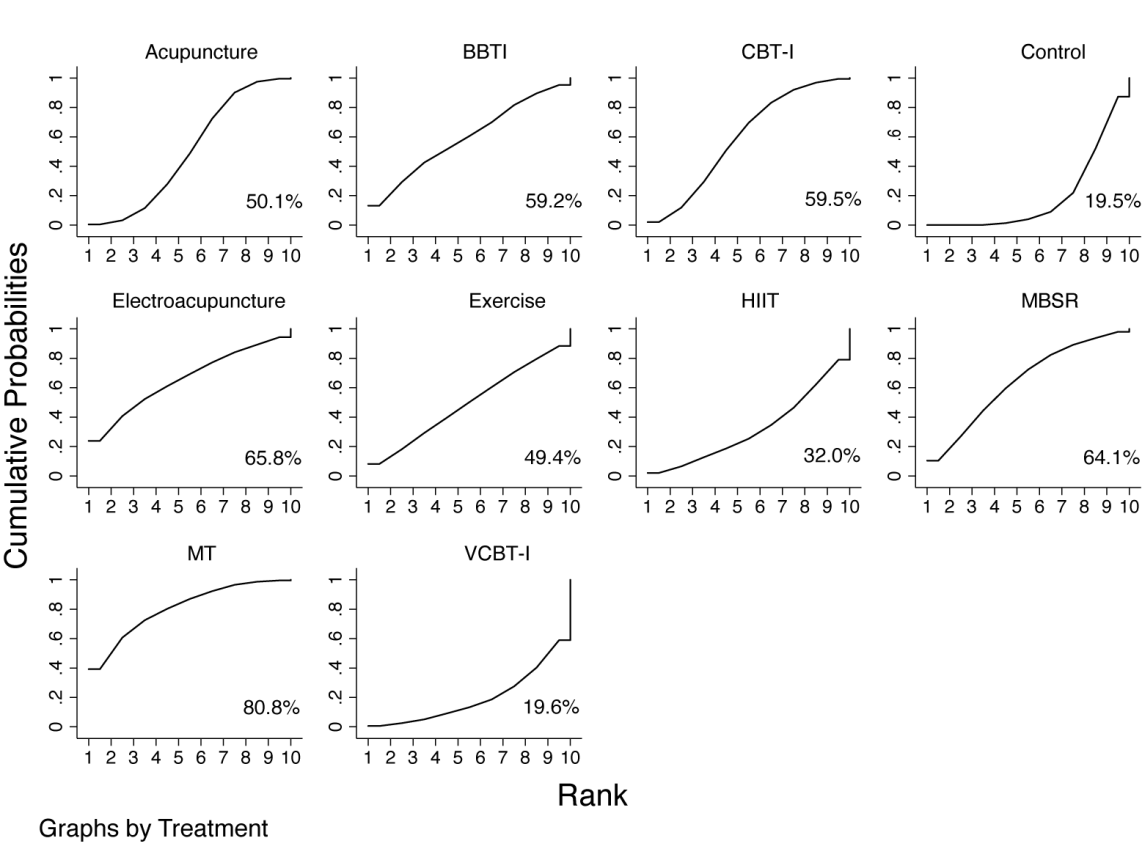


**Figure S6 SUCRA Ranking Plot for PSQI**

**Table S1 Abbreviations and Full Names of Intervention Measures**

| Non-drug interventions | Abbreviation |
| --- | --- |
| Cognitive behavioral therapy for insomnia | CBT-I |
| Mindfulness-based stress reduction | MBSR |
| Brief Behavioral Treatment for Insomnia | BBTI |
| Electroacupuncture | Electroacupuncture |
| Acupuncture | Acupuncture |
| Internet-delivered cognitive-behavioral therapy for insomnia | VCBT-I |
| Professionally administered Cognitive behavioral therapy for insomnia | PCBT-I |
| Mindfulness-based cognitive therapy | MBCT |
| Exercise | Exercise |
| High-intensity interval training | HIIT |
| Massage therapy | MT |

**Table S2 SUCRA Rankings for Non-Pharmacological Interventions Based on ISI and PSQI**

| Treatment | SUCRA(ISI) | PrBest | MeanRank | Treatment | SUCRA(PSQI) | PrBest | MeanRank |
| --- | --- | --- | --- | --- | --- | --- | --- |
| Acupuncture | 38.90% | 0.0 | 7.1 | Acupuncture | 50.10% | 0.4 | 5.5 |
| BBTI | 41.80% | 1.6 | 6.8 | BBTI | 59.20% | 13.2 | 4.7 |
| CBT-I | 55.90% | 0.1 | 5.4 | CBT-I | 59.50% | 2 | 4.6 |
| Control | 16.10% | 0.0 | 9.4 | Control | 19.50% | 0 | 8.2 |
| Electroacupuncture | 92.20% | 64.4 | 1.8 | Electroacupuncture | 65.80% | 23.9 | 4.1 |
| Exercise | 24% | 0.2 | 8.6 | Exercise | 49.40% | 8.2 | 5.6 |
| HIIT | 45.60% | 2.8 | 6.4 | HIIT | 32% | 2 | 7.1 |
| MBCT | 64.10% | 8.9 | 4.6 | MBSR | 64.10% | 10.4 | 4.2 |
| MBSR | 37.90% | 0.3 | 7.2 | MT | 80.80% | 39.2 | 2.7 |
| PCBT-I | 78.40% | 18.1 | 3.2 | VCBT-I | 19.60% | 0.6 | 8.2 |
| VCBT-I | 55.20% | 3.6 | 5.5 |  |  |  |  |
